# Autonomic Balance Differences Through Heart Rate Variability Between Adults with and Without Chronic Low Back Pain

**DOI:** 10.3390/healthcare13050509

**Published:** 2025-02-26

**Authors:** Carlos Fernández-Morales, Luis Espejo-Antúnez, Manuel Albornoz-Cabello, Ángel Rufino Yáñez-Álvarez, María de los Ángeles Cardero-Durán

**Affiliations:** 1 Department of Medical-Surgical Therapy, Faculty of Medicine and Health Sciences, University of Extremadura, 06006 Badajoz, Spain; carlosfm@unex.es (C.F.-M.); mcarderod@unex.es (M.d.l.Á.C.-D.); 2 Department of Physiotherapy, University of Seville, 41009 Seville, Spain; malbornoz@us.es (M.A.-C.); ayalvarez@us.es (Á.R.Y.-Á.)

**Keywords:** chronic low back pain, heart rate variability, autonomic nervous system

## Abstract

Background: Chronic pain has been reported as one of the leading causes of disability in the world, being associated with a potential impact on autonomic balance. Objective: The aim was to compare sympathetic and parasympathetic activity through heart rate variability (HRV) between adults with and without chronic low back pain (CLBP). Methods: An observational study was conducted in which HRV parameters were recorded using time-domain measures—root mean square of successive differences between consecutive RR intervals (rMSSD), minimum and maximum heart rate variability (Min HR and Max HR), and mean heart rate (Mean HR)—and nonlinear measures—Poincaré plot indices SD1 and SD2, Stress Score (SS), and sympathetic/parasympathetic ratio (S:PS). Results: The results showed statistically significant differences between groups (*p* < 0.05), with higher parasympathetic activity parameters in the group of healthy subjects (rMSSD: *p* < 0.001; SD1: *p* = 0.030) and higher sympathetic activity in the CLBP group (SD2, SS, and S:PS ratio: *p* < 0.001). All parameters showed large effect sizes. Conclusions: These findings show the association between autonomic balance mechanisms and pain regulation in adults with CLBP.

## 1. Introduction

Chronic low back pain (CLBP) forms one of the leading causes of disability worldwide and expenditure on health care. It presents a series of emotional and psychological symptoms and associated functional limitations [1]. Previous studies have associated the regulatory mechanisms of musculoskeletal pain with the function of the sympathetic and parasympathetic nervous systems [2]. These mechanisms involve the nervous system, affecting its scope, namely peripheral receptors, the spinal cord, and certain brain structures such as the prefrontal cortex and the limbic system [3]. Thus, its changes lead to dysfunctional adaptive regulation of the autonomic nervous system (ANS), which controls many essential functions, e.g., heart rate and blood pressure [4,5]. Such dysfunction is associated with maladaptive automatic responses that perpetuate the experience of pain [2]. In patients with chronic pain, these responses have been characterized by the hyperactivity of the sympathetic system and a reduction in parasympathetic modulation [5,6]. Specifically, this has also been reported in patients with low back pain. Telles et al. [7] observed an increase in vagal activity dominance after 3 months of supervised therapeutic exercise in patients with chronic low back pain associated with altered alignment of intervertebral disks.

Heart rate variability (HRV) is a valid, reliable, and non-invasive measure to evaluate ANS function [8,9]. HRV reflects the interaction between parameters of sympathetic and parasympathetic activity in the autonomic nervous system (ANS), indirectly reporting the autonomic mechanisms that regulate body homeostasis [8,10]. Among chronic pain conditions, the role of HRV in CLBP and its specific impact on disease activity have emerged as an important area of research [6,11]. In this sense, an increase in HRV is considered an indicator of a healthy autonomic response, while a reduction is associated with pathological conditions such as cardiovascular diseases, psychological disorders, or chronic pain [2,6]. In recent years, HRV has been used in clinical settings, workplace contexts, and experimental research. It has been considered an indicator of autonomic dysfunctions as well as systemic bodily responses in subjects with and without pain [8,12,13,14,15].

Despite the association between decreased vagal activity (reduced HRV), which is primarily related to parasympathetic modulation and altered endogenous descending inhibitory pain mechanisms in subjects with fibromyalgia, chronic cervical and low back pain, or chronic fatigue syndrome, among others [2,5,16], studies analyzing autonomic control through HRV in adults with CLBP are limited. Recent studies suggest that parasympathetic activity and autonomic regulation are involved in the modulation of pain responses through neurophysiological pathways [12,17].

In addition, authors such as Tracy et al. [2] and Koenig et al. [5] have focused on assessing psychophysiological parameters in individuals with chronic pain [2,5]. Equally, previous studies have hypothesized about the clinical behavior of certain parameters [7,17,18]; however, as far as the authors know, there are no studies that compare the values obtained with a sample of individuals in the absence of disease [2,5]. This prevents the clear identification of any differences in autonomic regulation between individuals with and without persistent pain.

Based on this evidence, we hypothesized that adults with CLBP would exhibit increased sympathetic activity (as indicated by higher SD2 and Stress Score) and decreased parasympathetic activity (as indicated by lower rMSSD and SD1) compared to adults without CLBP, resulting in an imbalance in autonomic regulation as measured by HRV. For this, the aim was to compare sympathetic and parasympathetic activity through HRV between adults with and without chronic low back pain (CLBP).

## 2. Materials and Methods

### 2.1. Study Design

This was a cross-sectional study. This study was supervised by the Institutional Ethics Committee of CEI University Hospital Virgen Macarena and Virgen del Rocio, with ethics approval number 1591-N-16, and was registered in ClinicalTrials.gov (NCT06760390). The study was performed in accordance with the Strengthening the Reporting of Observational Studies in Epidemiology (STROBE) statement [19]. Regarding ethical procedures, all participants signed an informed written consent form to participate in this study.

### 2.2. Participants

The study included two groups: a group of subjects without pain and a group of subjects with CLBP. The recruitment period went from 1 to 30 November 2020. The inclusion criteria for subjects with low back pain were (i) age between 18 and 65 years [5]; (ii) experiencing CLBP for ≥3 months (diagnosed by a physician) [20]; (iii) patients suffering from pain between the costal margins and the inferior gluteal folds with or without referred pain to the leg were included, provided that they scored at least 3/10 on the Numerical Pain Rating Scale [20,21]. The exclusion criteria were (i) any uncontrolled neurological or cardiac disorder [9]; (ii) chronic fatigue syndrome, fibromyalgia, or complex regional pain syndrome; (iii) regular use of medications that could affect the autonomic nervous system or pain perception, such as opioids, antidepressants, benzodiazepines, anti-inflammatory drugs, and beta-blockers, during the two weeks prior to the study [17]; and (iv) body mass index (BMI) equal to or greater than 30 kg/m^2^.

The subjects without pain selected had to meet the following criteria: (i) absence of any chronic or acute pathology; (ii) not be pregnant, including up to 6 months postpartum; (iii) not be under regular medication that could alter the ANS [17]; and (iv) have had no low back pain in the last six months.

**Figure 1:**
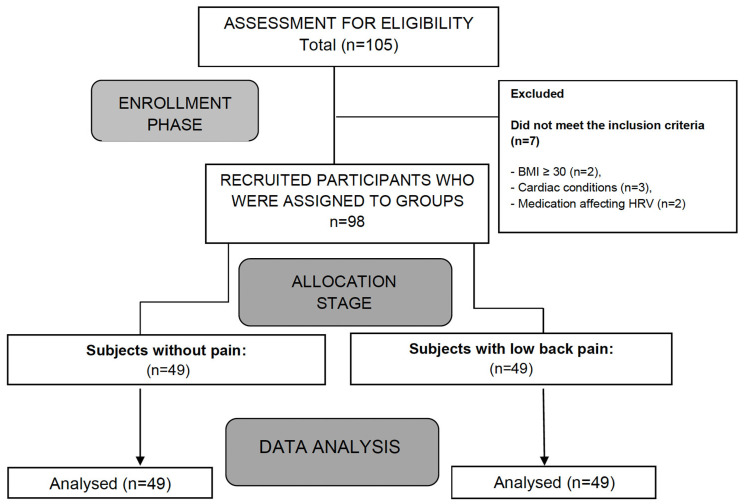
Flowchart of participant recruitment.

Participants were asked to avoid any medication or physiotherapy treatment in the last 24 h before the assessments; they were also informed about the nature of the study, and written informed consent was obtained from all participants prior to the first assessment. Figure 1 provides a flowchart of the subject recruitment carried out during the study.

### 2.3. Outcome Measures

Heart rate variability (HRV): This parameter has been recognized as an indicator of change detection in autonomic modulation using the R-R intervals recorded with a Firstbeat Bodyguard^®^ device (Firstbeat Technologies, Jyväskylä, Finland). R-R intervals represent the time elapsed between two consecutive R peaks in the electrocardiogram (ECG), which correspond to ventricular depolarization. These intervals are used to assess the variability in heart rate, reflecting the balance between sympathetic and parasympathetic activity [9]. Data collection was conducted over a 10 min period. The collected data were transferred to a computer using Firstbeat Uploader Plugin (Firstbeat Technologies Oy, Jyväskylä, Finland) and analyzed with Kubios^®^ HRV software (v.2.1) (University of Eastern Finland, Kuopio, Finland) [22].

The HRV methodology adhered to the recommendations of the Task Force of the European Society of Cardiology and the North American Society of Pacing and Electrophysiology [9] and incorporated guidelines from previous studies using the same equipment [14,15]. The HRV assessment employed the Poincaré plot, a validated tool for capturing nonlinear trends in R-R interval variability [23,24], and this assessment is recognized for its ability to provide insights into both sympathetic and parasympathetic activity under diverse conditions, including chronic low back pain [6,8,15].

HRV was assessed using both time-domain and nonlinear measures. The following parameters were analyzed:

Time-domain measures:

- Mean HR (bpm): this parameter corresponds to the average interval between two consecutive R peaks on the ECG, reflecting the mean heart rate over the recording period [10,23].
- rMSSD (root mean square of successive differences) (ms): This parameter represents the square root of the average of the sum of the squared differences between normal adjacent RR intervals. It reflects short-term variability and is directly associated with parasympathetic nervous system (PNS) activity. Higher rMSSD values indicate greater parasympathetic modulation [10,23].
- Min HR and Max HR (bpm): these parameters indicate the minimum and maximum heart rate, respectively, observed during the recording period [10,23].

Nonlinear measures:

- SD1 (ms): This parameter reflects the short-term variability in the nonlinear range of HRV and is considered an indicator of parasympathetic activity. It is derived from the Poincaré plot, which graphically represents the relationship between successive RR intervals [10,23].
- SD2 (ms): This parameter reflects long-term variability in the nonlinear range of HRV. It is also derived from the Poincaré plot and is considered an inverse indicator of parasympathetic activity, reflecting long-term changes in RR intervals [10,23].
- Stress Score (SS) (ms): This index, described by Naranjo-Orellana et al. [25], is calculated as the inverse of SD2 multiplied by 1000. It is directly proportional to sympathetic activity in the sinus node, with higher values indicating increased sympathetic dominance [10,23].
- Sympathetic/parasympathetic ratio (S:PS): This ratio, also described by Naranjo-Orellana et al. [25], is calculated as the quotient of SS and SD1. It reflects the balance between sympathetic and parasympathetic activity, with higher values indicating a predominance of sympathetic activity.

All measurements followed standardized HRV assessment guidelines to ensure methodological consistency [8,9]. The evaluations were conducted in the facilities of the Faculty of Medicine and Health Sciences at the University of Extremadura. Participants were instructed to fast overnight and to abstain from alcohol, caffeine, nicotine, and strenuous physical activity for at least 24 h before testing, as well as to ensure they got a full night’s rest. HRV was recorded in both groups while laying in a prone position. Prior to data collection, a mandatory 10 min resting period in a quiet environment was implemented to ensure autonomic stabilization.

### 2.4. Sample Size

The sample size was estimated using G*Power 3.1.9.7 software (Düsseldorf, Germany). For a *t*-test comparing means between two independent groups (subjects without pain vs. subjects with CLBP), the following parameters were used: an effect size (d) of 0.60, an alpha level of 0.05, and a statistical power of 0.80. This calculation resulted in a minimum sample size of 45 participants per group. To address the possibility of incomplete data, the sample size was increased by at least 5%, resulting in a final sample of 49 participants per group, totaling 98 participants.

### 2.5. Statistical Analysis

A descriptive analysis was performed for all quantitative variables, presenting the mean and standard deviation for each group. To compare the two groups, Student’s *t*-test for independent samples was applied, as the normality assumption was verified using the Shapiro–Wilk test. Furthermore, the effect size was calculated through Cohen’s d coefficient. A value above 0.8 was considered high, around 0.5 was considered moderate, and lower than 0.2 was considered low [26].

Additionally, a univariate analysis of covariance (ANCOVA) was conducted to compare the HRV-related variables between groups, adjusting for body weight (kg) and body mass index (BMI) as covariates. Adjusted means and 95% confidence intervals (CIs) were reported for each variable. The significance of the group effect was assessed using the F-statistic, and partial eta-squared (η_p_^2^) was calculated to estimate the effect size, with values above 0.06 considered large, around 0.01 moderate, and below 0.01 small [26].

The significance level was set at *p* < 0.05. The collected HRV data were exported to an Excel spreadsheet and structured by participant ID, time-domain variables (Mean HR, Min HR, Max HR, rMSSD), and nonlinear variables (SD1, SD2, Stress Score, and S:PS ratio). Data preprocessing included checking for missing values and ensuring signal integrity before statistical analysis. Data analysis was performed using SPSS Statistics, version 22.0 (SPSS Inc., Chicago, IL, USA).

## 3. Results

A total of 98 adults with and without CLBP participated in the study. They were divided into two groups: subjects without pain (n = 49) and subjects with CLBP (n = 49). The baseline characteristics of the sample are presented in Table 1. No statistically significant differences were found between groups in variables such as age (*p* = 0.653) and height (*p* = 0.283). However, statistically significant differences were observed in weight (*p* < 0.001) and body mass index (BMI; *p* < 0.001).

**Figure 2:**
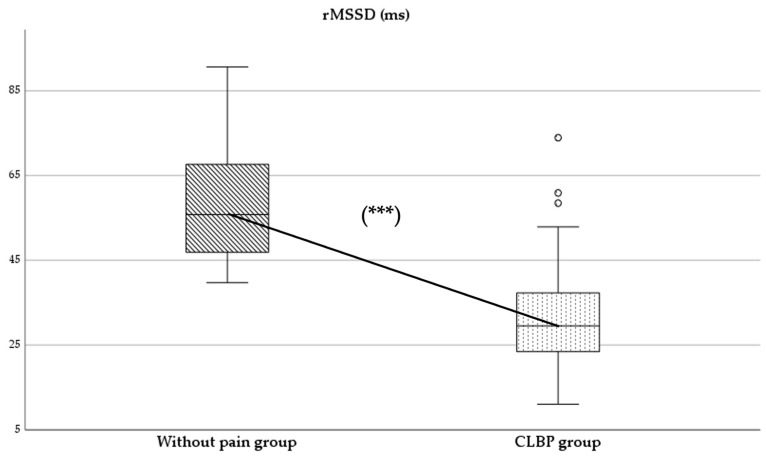
Comparison of rMSSD values between without-pain group and CLBP group. *** indicates statistically significant differences with *p* < 0.001.

**Figure 3:**
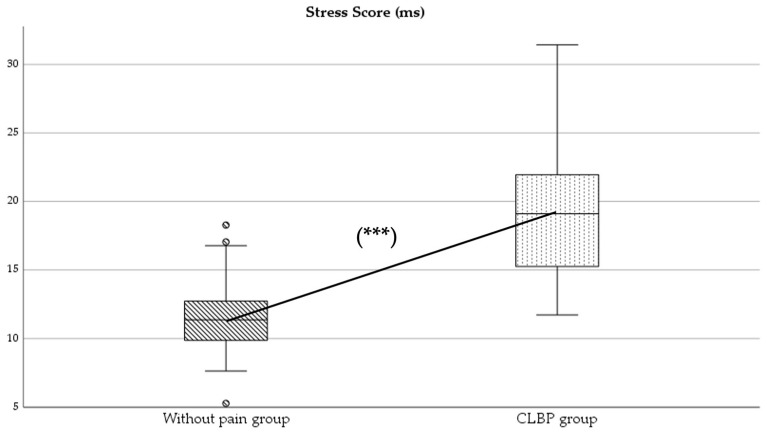
Comparison of Stress Score (SS) values between without-pain group and CLBP group. *** indicates statistically significant differences with *p* < 0.001.

Regarding parasympathetic activity parameters, both rMSSD and SD1 showed significantly higher values in the group of subjects without pain compared to those with CLBP (*p* < 0.05), with large (*d* = 2.0) (Figure 2) and moderate (*d* = 0.4) effect sizes, respectively. As for sympathetic activity parameters, while SD2 presented higher values in subjects without pain (*p* < 0.001), with a large effect size (*d* = 1.8), the SS reported statistically higher results in the CLBP group (*p* < 0.001; *d* = 1.6) (Figure 3). Finally, the sympathetic/parasympathetic ratio (S:PS) presented higher values in the CLBP group (*p* < 0.001), with a large effect size (*d* = 1.4).

A univariate analysis of covariance (ANCOVA) was performed to compare HRV-related variables between groups, adjusting for weight (kg) and body mass index (BMI) as covariates. The adjusted means for Min HR were higher in the CLBP group (65.24, IC 95%: 62.07–68.41) compared to subjects without pain (62.16, IC 95%: 58.99–65.33), although this difference did not reach statistical significance (F_1,94_ = 1.67, *p* = 0.199, η_p_^2^ = 0.017). Regarding Max HR, the adjusted means were significantly higher in the subjects in the without-pain group (99.96, IC 95%: 95.16–104.75) compared to the CLBP group (89.29, IC 95%: 84.50–94.08), with a statistically significant difference (F_1,94_ = 8.79, *p* = 0.004, η_p_^2^ = 0.086). For Mean HR, no statistically significant differences were found between the groups, with adjusted means of 74.77 (IC 95%: 71.12–78.42) in the without-pain group and 74.49 (IC 95 %: 70.82–78.14) in the CLBP group (F_1,94_ = 0.010, *p* = 0.920, η_p_^2^ = 0.000). For rMSSD, the adjusted means were higher in subjects without pain (58.26, 95% CI: 54.10–62.41) compared to the CLBP group (58.26, IC 95%: 54.10–62.41) compared to the CLBP group (32.35 ms, IC 95%: 28.14–36.44), showing a statistically significant difference (F_1,94_ = 69.50, *p* < 0.001, η_p_^2^ = 0.425).

Regarding nonlinear variables, the adjusted means showed statistically significant differences between groups (*p* < 0.05). The adjusted means for SD1 were higher in the group of subjects without pain compared to the group with CLBP (41.16 [IC 95%: 35.84–46.48] vs. 31.94 [IC 95%: 26.61–37.26]; F_1,94_ = 5.35, *p* = 0.023, η_p_^2^ = 0.054). Similarly, the adjusted means for SD2 were higher in the group of subjects without pain (90.41, [IC 95%: 84.56–96.26] vs. (55.10, [IC 95%: 49.25–60.95]); F_1,94_ = 64.69, *p* < 0.001, η_p_^2^ = 0.408). On the other hand, the comparison of adjusted means between groups for SS (Stress Score) (12.22, [IC 95%: 10.87–13.57]) vs. (19.34, [IC 95%: 17.98–20.69]) reported a statistically significant reduction in the group of subjects without pain (F_1,94_ = 49.18, *p* < 0.001, η_p_^2^ = 0.343). Finally, the comparison between groups for the S:PS ratio (subjects without-pain group: 0.31, IC 95%: 0.18–0.44 vs. CLBP group 0.91, IC 95%: 0.78–1.04) also showed significantly lower values in the subjects without pain (F_1,94_ = 36.81, *p* < 0.001, η_p_^2^ = 0.281).

## 4. Discussion

The aim of this study was to compare sympathetic and parasympathetic activity through heart rate variability (HRV) between adults with and without CLBP.

HRV has been considered an important diagnostic measure of neurogenic homeostatic regulatory capacity in subjects with low back pain [6]. The statistically significant differences found between groups in parameters of parasympathetic and sympathetic activity might indicate a possible alteration in autonomic balance in the CLBP group (Figure 2 and Figure 3).

The findings of this study suggest a potential bidirectional relationship between CLBP and autonomic dysfunction. On one hand, persistent pain may lead to sustained sympathetic hyperactivity and reduced parasympathetic modulation, as observed in our results. This could be due to the activation of stress pathways and the inhibition of descending pain inhibitory mechanisms, which are modulated by the autonomic nervous system [2,5,16]. These results are consistent with previous studies that associate decreased heart rate variability with other chronic pain conditions such as fibromyalgia, temporomandibular disorders, chronic cervical and shoulder pain, and chronic low back pain, among others [2,5,6,11]. On the other hand, autonomic dysfunction, particularly sympathetic dominance, may exacerbate pain perception by promoting systemic inflammation, increasing muscle tension, and altering pain processing at the central level [6,11]. In this regard, previous studies have highlighted the relationship between decreased rMSSD and SD1 with states of sympathetic hyperactivity [2,5,6].

The homogeneous selection of participants with CLBP helped minimize variability in the results by limiting the multifactorial and contextual factors characteristic of other chronic pain conditions, allowing for a more accurate assessment of the ANS in this population [27,28].

Time-domain measures:

The comparison between groups showed a significant reduction in parasympathetic activity in patients with CLBP (difference: rMSSD: −27.41 ms; SD1: −7.96 ms). The study results were consistent with the scientific evidence available for other chronic pain conditions [2,5,18]. In this sense, Nunan et al. [29] reported cut-off values for the rMSSD parameter, set at 45 ± 15 ms for healthy adults, which could be higher in younger and physically active populations. Our results were consistent with these authors, finding differences between both groups around these normative values (CLBP group: 31.57 ± 13.04 ms vs. group without pain: 58.98 ± 14.75 ms) [29]. The results obtained in the group without pain were slightly higher. This finding could be explained by the mean age of the sample, which was lower than that reported by Nunan et al. [29] (37.71 ± 14.85 years) (Table 1). In contrast to refs. [2,5,16], the CLBP group showed an rMSSD parameter slightly lower than those reported by Koening et al. [5] in patients with chronic pain (31.57 ± 13.04 ms vs. 35.48 ± 12.82 ms). Future studies are needed to describe reference values for autonomic activity in chronic pain conditions with higher prevalence in the population.

Regarding Mean HR, Min HR, and Max HR, no statistically significant differences were found, except for a slight trend toward lower variability amplitude in the CLBP group. The absence of changes could be due to the fact that these parameters show lower variability and sensitivity to changes in autonomic modulation compared to others like rMSSD [29].

Nonlinear measures:

Statistically significant differences were found between the groups, characterized by an increase in sympathetic activity in subjects with CLBP (Table 1). Specifically, SD1 and SD2 from the Poincaré plot, indicators of short- and long-term variability, respectively [10], showed a significant reduction in the CLBP group (Table 1). The reduction in SD1 is associated with decreased parasympathetic activity [10]. These findings align with previous studies that associate these alterations in homeostatic regulatory mechanisms with states of central sensitization and chronic stress [2,7]. The fact that the effect size for SD1 was low (*d* = 0.4) could be due to the position in which the measurements were taken, as lower sympathetic dominance appears to be associated with static positions like the supine position, especially in young populations [30,31].

On the other hand, the Stress Score (SS), an indicator of sympathetic activity [25], showed significantly higher values in individuals with CLBP (Figure 3). This finding is consistent with the predominance of sympathetic activity in populations with pain [2,5,6].

An S:PS ratio of ≥0.3 at rest reflects a predominance of sympathetic activity or a reduction in the recovery capacity of parasympathetic activity [15,25]. In both groups, measures equal to or greater than 0.3 were observed [25]. This result could be explained by three reasons: (i) the recording of variables exceeded the minimum required time (5 min) according to international recommendations [9]; (ii) the supine resting position influences autonomic modulation [30,31]; and (iii) the interpretation of pain behavior in the group of individuals with pain, due to the wide variety of self-reported symptoms within the same clinical entity (CLBP) [16].

Although weight (kg) and BMI showed statistically significant differences between groups (73.19 ± 8.61 vs. 82.30 ± 14.52; *p* < 0.001 and 22.85 ± 1.86 vs. 25.27 ± 2.95; *p* < 0.001, respectively), the differences found in all parameters of parasympathetic and sympathetic activity remained after adjusting for both anthropometric variables using a univariate analysis of covariance (ANCOVA). Recently, Bigand et al. [32] and Miranda et al. [33] have associated chronic pain with increased body weight, likely due to reduced physical activity and the side effects of medication or psychological stress that often accompany this condition. Despite this, the results found in the present study do not show that the body weight of individuals in either group influences any of the parameters of parasympathetic and sympathetic activity. Considering this, future studies are needed to establish follow-up periods to monitor these variables in the medium and long term in individuals with CLBP.

In conclusion, the results obtained support the hypothesis that chronic pain is not limited to a sensory experience but encompasses other aspects, such as autonomic regulatory capacity [34]. The differences observed in autonomic control could, in part, be a manifestation of the interaction between the central and peripheral nervous systems, exacerbated by psychological factors such as anxiety and catastrophizing [17].

### 4.1. Clinical Implications

HRV can be considered an objective and useful measure for detecting autonomic imbalances in individuals with CLBP [6,11]. The values of the different HRV parameters recorded could be used as a reference when analyzing the impact of therapeutic interventions aimed at restoring autonomic balance [6,15,35].

### 4.2. Limitations

The main limitations of the study were as follows: (i) The data analysis focused on time-domain and nonlinear metrics. Future studies should include frequency-domain variables to broaden comparisons with other studies [6]. (ii) The sample was composed of adults without significant comorbidities, limiting the extrapolation of the findings to other populations, such as adolescents or older adults, and (iii) pain intensity was assessed as part of the inclusion criteria but not measured immediately before the HRV assessment. Future studies should consider incorporating real-time pain measurements to better understand the dynamic relationship between pain and autonomic regulation.

## 5. Conclusions

There are differences between adults with and without CLBP in terms of both parasympathetic (rMSSD and SD1) and sympathetic activity (SD2 and SS), as measured by HRV. Additionally, the S:PS ratio highlights an imbalance in autonomic regulation, with a predominance of sympathetic activity in subjects with CLBP.

## Figures and Tables

**Table 1:** Baseline characteristics and between-group comparison of subjects without pain and individuals with chronic low back pain.

Variable	Total Sample (n = 98)	Without-Pain Group (n = 49)	CLBP Group (n = 49)	*p*-Value *	*d*
Age (years)	38.41 ± 15.18	37.71 ± 14.85	39.10 ± 15.63	0.653	-
Height (cm)	178.06 ± 6.66	178.92 ± 7.10	177.49 ± 5.96	0.283	-
Weight (kg)	75.15 ± 12.77	73.19 ± 8.61	82.30 ± 14.52	<0.001 **	0.8
BMI (kg/m^2^)	23.52 ± 2.73	22.85 ± 1.86	25.27 ± 2.95	<0.001 **	0.9
Min HR (bpm)	63.07 ± 11.15	61.26 ± 8.45	66.14 ± 12.87	0.029 *	0.4
Max HR (bpm)	82.53 ± 12.70	100.35 ± 16.54	88.90 ± 15.27	0.001 *	0.7
Mean HR (bpm)	74.52 ± 12.21	74.25 ± 11.51	75.01 ± 13.05	0.763	-
rMSSD (ms)	45.27 ± 19.53	58.98 ± 14.75	31.57 ± 13.04	<0.001 **	2
SD1 (ms)	32.03 ± 14.81	40.53 ± 7.92	32.57 ± 24.02	0.030 *	0.4
SD2 (ms)	72.75 ± 26.88	90.67 ± 24.27	54.84 ± 13.61	<0.001 **	1.8
SS (ms)	15.78 ± 5.72	12.10 ± 3.92	19.46 ± 5.27	<0.001 **	1.6
S:PS ratio	0.61 ± 0.53	0.30 ± 0.14	0.92 ± 0.60	<0.001 **	1.4

Data are presented as mean ± SD. BMI = body mass index; CLBP: chronic low back pain; Min HR = minimum heart rate (bpm); Max HR = maximum heart rate (bpm); Mean HR = average heart rate (bpm); rMSSD = root mean square of successive differences (ms); SD1 = transversal axis of Poincaré plot (ms); SD2 = longitudinal axis of Poincaré plot (ms); SS = stress score (inverse of SD2 × 1000); S:PS ratio = quotient of SS and SD1. * indicates statistically significant between-group differences (*p* < 0.05); ** indicates statistically significant differences with *p* < 0.001; *d* = Cohen’s d.

## Data Availability

The data presented in this study are available on request from the corresponding author.
